# Touch Hand 4.5: low-cost additive manufacturing prosthetic hand participated in Cybathlon 2020 ARM discipline

**DOI:** 10.1186/s12984-022-01112-5

**Published:** 2022-11-30

**Authors:** Kashreya Moodley, Jode Fourie, Zaahid Imran, Clive Hands, William Rall, Riaan Stopforth

**Affiliations:** 1 Stopforth Mechatronics, Robotics Research Lab, University of KwaZulu-Natal, Durban, South Africa; 2 Advanced Engineering Design Group, Nelson Mandela University, Port Elizabeth, South Africa

**Keywords:** Prosthetic hand, Cybathlon, Touch Hand, Additive manufacturing, Low cost

## Abstract

**Background:**

The Touch Hand 4.5 is a highly customisable prosthetic hand, which features an optimised modular design of the Touch Hand 4. The Touch Hand team has developed a low-cost prosthetic hand, which has been built using an additive manufacturing process. The functionality and features are discussed that are crucial for amputees.

**Methods:**

This paper documents the design and integration of the Touch Hand 4.5 to be used in the Cybathlon 2020 event as well as the development of the mechanical structure of the hand, socket, electronics and control system utilized. The Touch Hand 4.5 was designed and continuously optimized, with the goal to achieve the tasks in the Cybathlon 2020 event.

**Results:**

The performance and functionality of the Touch Hand 4.5 was tested on a global scale at the Cybathlon 2020. The device and technology were evaluated against the leading prosthetics and prototypes from around the world. A series of everyday tasks, as set by the Cybathlon event, were performed to determine the capabilities of the device, with the pinch grip, full grip, half grip, and a thumb grip. The Touch Hand team was the only team to complete the Haptic Box task in all three races, which comprised of the identification of objects without the aid of visual input or perception, with a duration between 100 and 120 s. The Breakfast task entailed completing a series of everyday breakfast tasks, such as cutting a loaf of bread, lighting a candle, opening a sugar packet, opening a plastic bottle and a jar, as well as opening a tin can with a can opener. This task was only completed in Race 3, with a duration of 132 s, due to a faulty equipment that was supplied.

**Conclusion:**

The first contribution that was achieved was the design and development of an additive manufactured hand and socket, considering the socket to have comfort, breathability and decreased irritability. The second contribution was the design optimisation with the linear actuator integration, for a multi-grip hand, which allowed for the pinch grip, full grip, half grip, and a thumb grip. Slippage prevention with grip force control system integration was also implemented.

*Trial registration number:* Ethical clearance certificate HCC/0161/011.

## Background

The Cybathlon is a multi-disciplinary event, which takes place on a global scale. The Cybathlon event has created an international platform, which serves to promote the research and development of powered prosthetics limbs and assistive technology [1]. The objective of this event is to highlight the functionality and performance of the powered prosthetic, by challenging the pilot to carry out a series of everyday tasks.

The six tasks used in the Cybathlon-ARM challenge, namely Breakfast, Laundry, Clean Sweep, Home Improvement, Haptic Box, and Stacking, were capable of testing a wide range of aspects related to the functionality and performance of the prosthetic arm [2]. These tasks are designed to challenge the hand strength and coordination of the device, the fine motor skills and the range of motion of the wrist and forearm. These tasks also ensure that aspects such as the size of the arm and the different grip types are easy to use in daily life are also thoroughly tested [2]. The Touch Hand 4.5, which is discussed in this paper, was designed and continuously optimized, with the goal to achieve the tasks in the Cybathlon 2020 event.

Touch Prosthetics have therefore developed an advanced low-cost prosthetic hand, which is available to the public and affordable for low-income households [3]. The Touch Hand 4.5 uses an additive manufacturing approach, which both reduces costs, and allows the device to be highly customisable to the user to ensure maximum comfort and functionality.

A common issue associated with the use of prosthetic limbs is the skin irritation caused by prolonged use of the prosthetic [4]. There are many side effects which can result from wearing an upper-limb prosthetic, such as heat rash, due to the increased skin temperature of the residual limb, contact dermatitis, in some cases, as well as painful ingrown hairs and blisters [4]. EMG noise can also be created by these mentioned factors.

A factor which must be carefully considered is the comfort of the prosthetic, the liner and the harness. In addition to these issues, the liner of the prosthetic can potentially serve as an incubator for bacteria [4]. Another common issue that many amputees suffer with is the lack of sensory feedback from the prosthetic device. Sensory feedback occurs when information is translated from the sensors to the device and converted into a series of electrical impulses, which are then translated by the interfaces of the device into a sensation. Although sensory feedback is vital to prosthetic device users, it forms part of a highly complex, multifaceted system, which has its limitations [5, 6].

Prosthetic arms are designed to meet the specific needs of the user. In order to satisfactorily meet the needs of the user, the prosthetic device should be able to carry out basic everyday tasks, provide sensory feedback to the user, perform a range of stable grips and provide the user with a reliable and efficient device [7]. There are many different types of prosthetics designed for upper limb amputees, such as passive prosthetics [8], body-powered prosthetics, which are operated using a body harness in combination with the upper body muscles and the pilot’s residual limb [8], myoelectric prosthetics [9], hybrid prosthetics [8] and Osseo-integrated prosthetics, which use intramedullary rodding in the residual bone [10]. The type of prosthetic chosen by the amputee depends on the comfort and functionality of the prosthesis, as well as the lifestyle, level of amputation and the specific needs of the user.

3D printing, or Additive Manufacturing, is a cost-effective method, which allows for the development of a highly customisable prosthetic device. 3D printing could be time consuming, but it is still faster than creating moulds for injection moulding, when developing customized products, such as the prosthetic socket that would be attached to an amputee. This method is therefore commonly used to manufacture various components within the device in order to create a cost effective, highly customisable prosthetic, with a decreased production time [11]. The manufacturing cost of the customized Touch Hand 4.5 socket was approximately US$ 500, compared to the manufacturing of conventional sockets which can cost more than US$ 3000 [12].

The Touch Hand team, which was the only team from the African continent, have set out to develop an innovative, yet affordable for their local demographic, solution which provides the user with a realistic and high-quality experience [3]. The Cybathlon 2020 event allowed the Touch Hand Team to thoroughly test the capabilities of the Touch Hand 4.5 design and served as a platform to draw up a comparison between their device and the other commercial and developmental prosthetics and prototypes participating in the event.

This paper looks specifically at the Breakfast and Haptic Box tasks, which were completed by the Touch Hand 4.5 in the Cybathlon 2020 event and how it compared with other prosthetic hands that participated. Even though there were some tasks that were possible to be done in the Clean Sweep and Stacking tasks., these are not discussed in detail, as the full stage was not completed due to the time limit for tasks to be performed.

The contributions of this research are the following:

Design and development of an additive manufactured hand and socket, considering the socket to have comfort, breathability and the decrease of irritability. A less irritable and breathable socket allows for a decrease in EMG noise and user-hand unity.

Design optimisation with the linear actuator integration, for a multi-grip hand, allowing for the pinch grip, full grip, half grip, and a thumb grip. Slippage prevention with grip force control system integration.

## Methods

The development of the design and construction of the Touch Hand 4.5, with respect to the mechanical structure, electronics and control and software is detailed in the following section. The Touch Hand 4.5 has been developed with a new design based on the design concepts which were implemented in the Touch Hand 4.

### Mechanical design

**Fig. 1:**
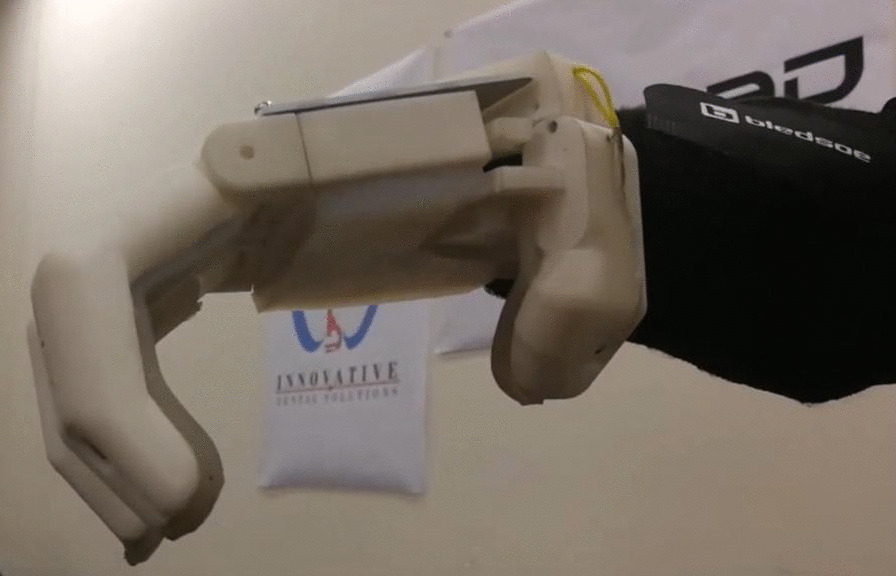
The Touch Hand 4 prototype

The Touch Hand 4 was constructed and developed as a prototype to be used in the Cybathlon 2020 event, yet optimisation was needed. The Touch Hand 4 prototype, which is the basis for optimisation described in this paper, is shown in Fig. 1.

The Touch Hand 4.5 features a redesign of the existing mechanical structure of the Touch Hand 4 [13]. With numerous tests of the Touch Hand 4 conducted by different amputees, the feedback allowed for the optimisation and improvements, specifically focussing on the functionality of the device. The development of the hand and socket structure of the Touch Hand 4.5 are detailed in the following section, which also took into account the ability for performing the tasks required by the Cybathlon 2020 event, to perform day-to-day activities. Therefore, the pinch grip, full grip, half grip, and a thumb grip were important to achieve. Even though a weight of 2kgs was being pursued to be picked up, it has been experienced that actuators rated for these forces outputted less than the desired value, so designs were considered for a weight of 5 kgs to be picked up. The size of the hand had to be minimalized, yet contain the actuators, and the weight had to be reduced as much as possible during the design. Aesthetics was important, yet users have indicated in the feedback that functionality is of higher priority.

#### Hand structure

**Fig. 2:**
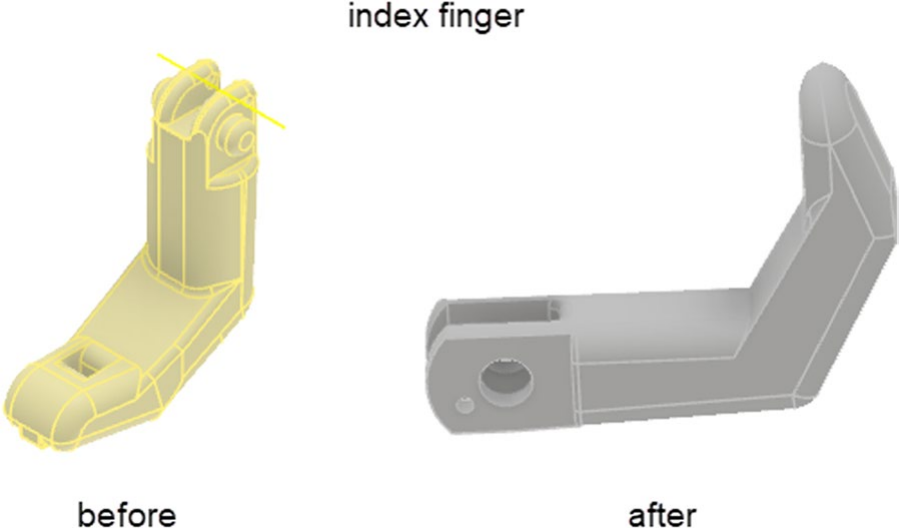
3D CAD model representing the index finger before and after the redesign

The challenge detected with the hand structure was the issue that existed between the digits and the knuckle structure. The CAD model representing the index finger before and after the redesign are represented in Fig. 2.

**Fig. 3:**
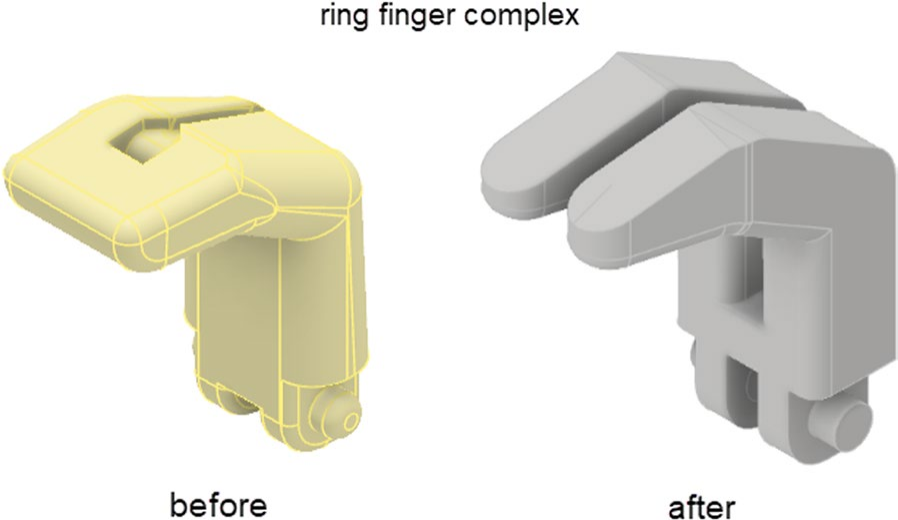
3D CAD model representing the ring finger complex before and after the redesign

The 'index finger' was made thinner, sleeker and pointier, which allowed the finger to be less bulky and ensured a finer grip when completely closed, as well as more compact relative positioning when the ‘fist’ is closed.

he secondary 'ring finger' complex was made to look aesthetically better by becoming two joined fingers and also made this component less bulky, whilst still being actuated with a single linear actuator. The angles between the fingers also had to be readjusted to accommodate proper fitment on closing. The redesigned ring finger complex can be seen in Fig. 3.

**Fig. 4:**
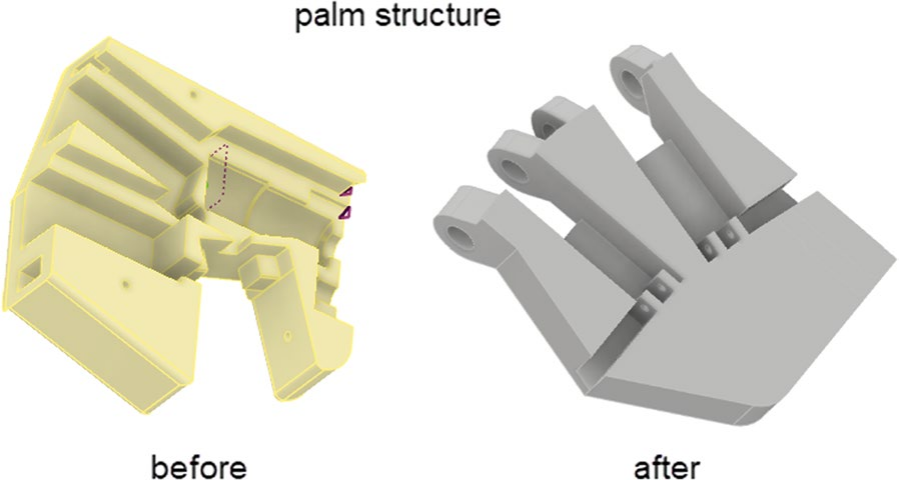
3D CAD model representing the palm structure before and after the redesign

Following the completion of the design of all the finger complexes, the palm structure was developed. The knuckles on the palm were rotated 45º downward to allow the fingers to close completely into a bicycle grip, and the orientation of the linear actuators were changed to sort out some of the play issues which were initially experienced. The comparative palm structure can be seen in Fig. 4.

he main initial problem with the functionality of the initial hand was the significant play of the 'fingers' on basic operation. The orientation of the linear actuators was changed to facilitate a smoother arc of movement and eliminate any excessive side thrust on the actuator shaft, by looking at various concepts of accommodating this motion.

**Fig. 5:**
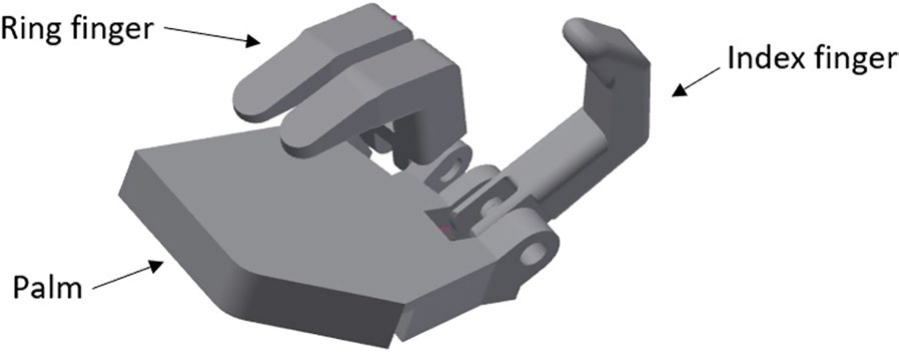
The intermediate assembly of the components, excluding the thumb

Further sculpting of the palm surface profile was carried out in order to produce a more effective gripping profile for the tasks that the hand would have to perform. Figure 5, shows the intermediate assembly of all the components, excluding the thumb component. The thumb orientation angle was changed and the thumb component itself had to be redesigned.

#### Orientation of linear actuators

The palm structure included the reorientation of the linear actuators to eliminate the play issues experienced.

The updated actuator angle was decided using a force analysis of the entire actuating mechanism. Actuonix PQ12-6-100 actuators were chosen as they delivered a 50N output force, which was sufficient for moving the finger mechanisms and provided enough force to hold day-to-day objects. The resultant force was obtained by means of Eq. (1).

1 $$\mathit{tan}\left(28^\circ \right)=\frac{Fy}{50}$$

$$Fy=50\times \mathit{tan}\left(28\right)=26.5 N$$

$$Fresult=\sqrt{{50}^{2}+F{y}^{2}}=56.588 N$$

where: Fy = Side thrust on actuator arm [N] Fresult = Resultant force in the actuating link [N].

**Fig. 6:**
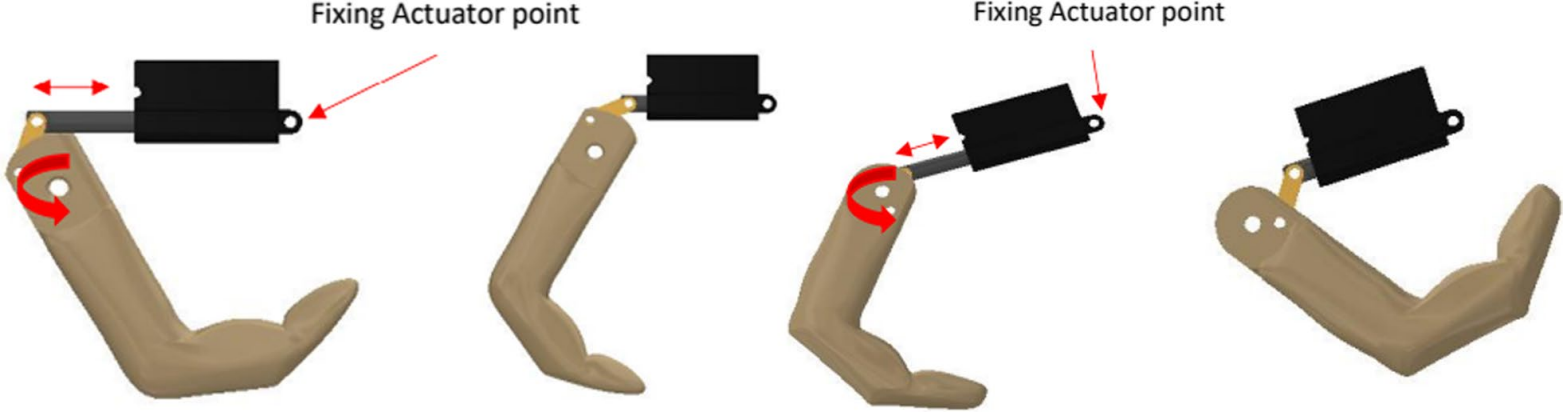
Two images on left shows the original actuation position, two images on right shows the improved actuation position

The original Touch Hand 4 design had the actuators horizontal to the palm of the hand, with the maximum side thrust being experienced when the actuator was fully extended and the fingers closed. This play caused excessive wear on the actuators and caused them to fail after some use. The mechanism was changed so that the fingers would be closed when the actuator was fully retracted and thus the side force would be experienced in this position rather than in the fully extended position, reducing the wear on the actuator arm, as seen in Fig. 6.

**Fig. 7:**
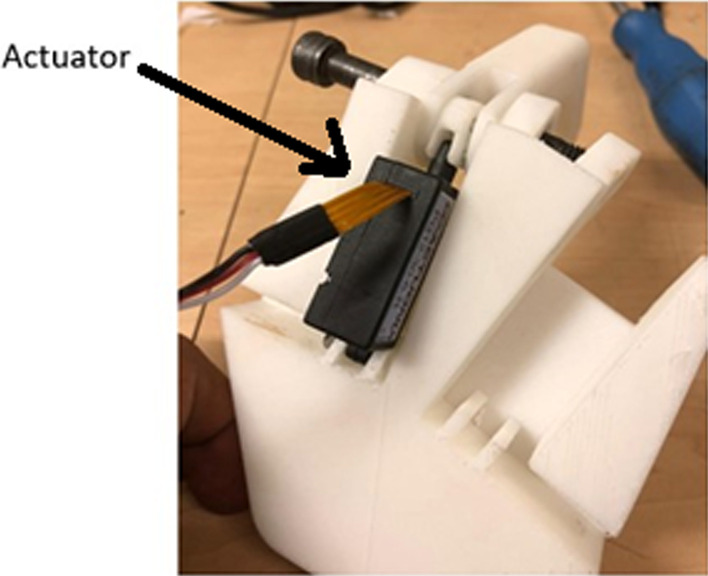
Positioning of linear actuator on the palm structure

An Acrylonitrile butadiene styrene (ABS) 3D printed assembly, which can be seen in Fig. 7, was carried out to test the functionality of the updated design. The printing of the Touch Hand 4.5 was done with a Selective Laser Sintering (SLS) printer.

Further sculpting of the palm was carried out to give the palm greater functionality in gripping objects, as well as giving the hand an aesthetically more pleasing and organic look. A further 3D print was carried out on the completed hand assembly using new actuator positions, knuckle links and pins. The final weight of the Touch Hand 4.5 was 4.7 kg.

**Fig. 8:**
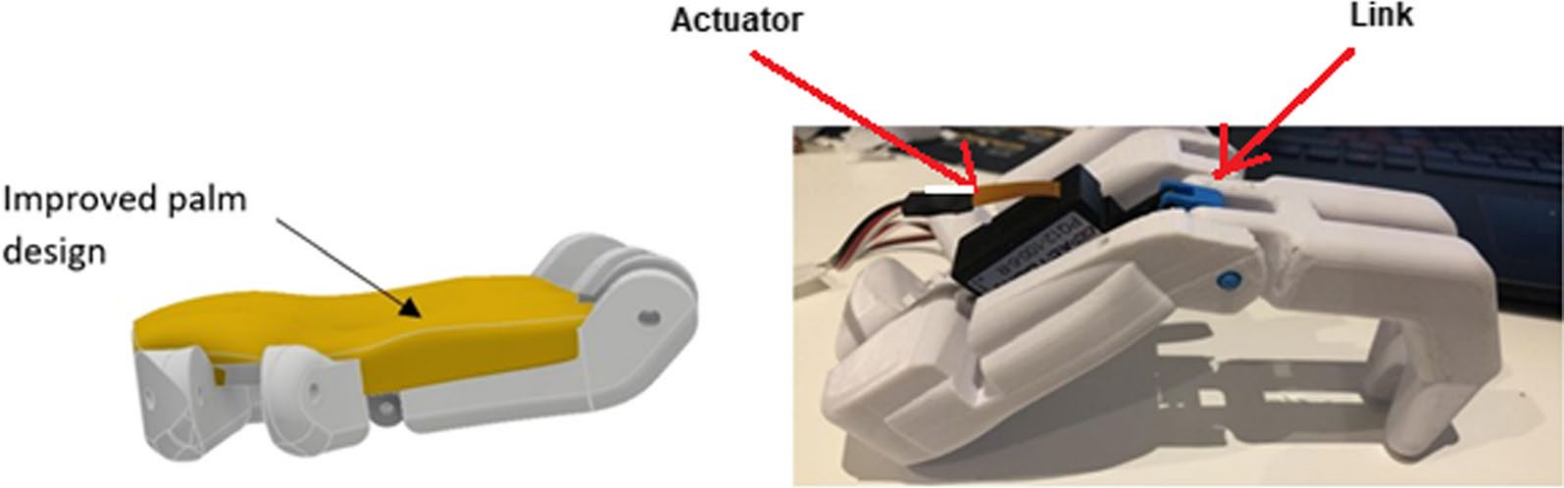
3D printed model showing the positioning of the linear actuator

The outcome was that the play issue on the actuator was solved, however a new problem was then identified. The allowing of the actuator to pivot on one end introduced too many movable points and no ground which caused the finger to move out of position, *i.e.,* a new type of play has been inadvertently introduced. This play was solved by removing the knuckle link, so that the actuator connected directly to the finger using a single link, correlating to a simplified bell crank system. Due to the removal of the knuckle link, the finger was not able to move to a fully crunched position due to issues with the mechanical advantage and pivot points of the link attaching actuator to finger. The positioning of the linear actuator can be seen connected to the updated palm structure in Fig. 8.

#### Silicone cover

**Fig. 9:**
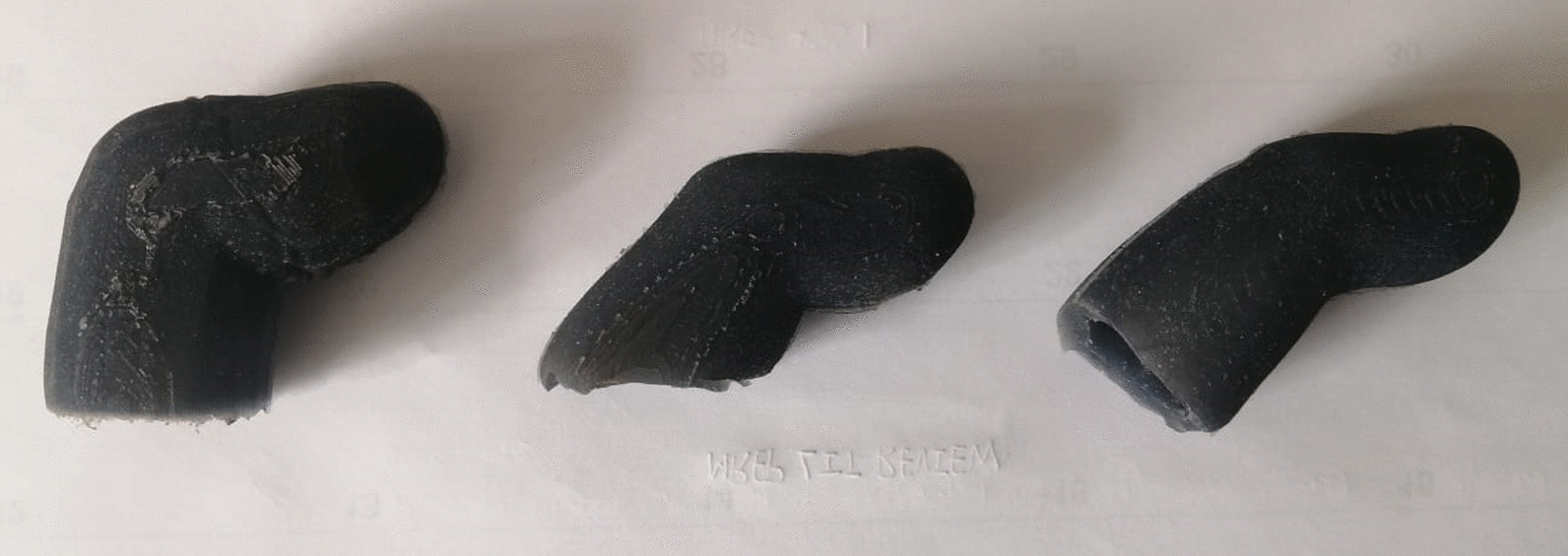
Silicone covers for the thumb, index and ring finger respectively

he hard plastic fingers of the 3D printed hand did not provide enough grip to allow the fingers to pick up smooth objects, such as a plastic cup, as a result, silicone slip-on covers were used on the fingertips of the hand. The silicone used was a 10-shore hardness silicone, which is very soft and has a skin-like texture. The silicone covers for the thumb, index and ring finger can be seen in the Fig. 9.

The silicone covers were manufactured using a casting process with split 3D printed moulds. The moulds were manufactured by creating a negative imprint of each finger in a block. Channels for ventilation and for pouring in the silicone were added to the mould. The block was then split in half to allow for the removal of the sleeve once it had cured.

#### Wrist structure

**Fig. 10:**
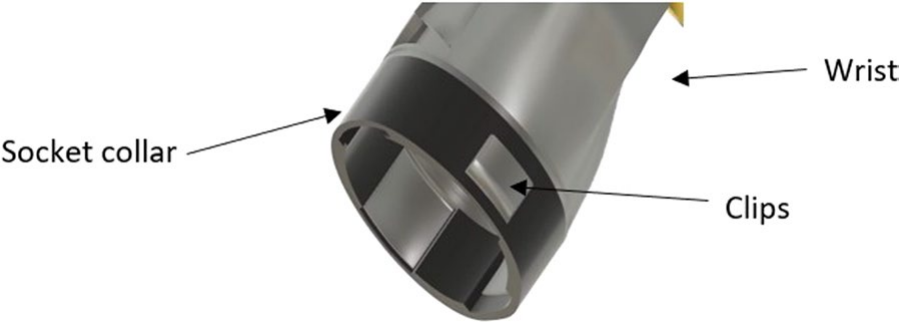
The snap fit clip clipped into the socket collar

The wrist-snap-fit-clip was used to connect the wrist to the socket of the Touch Hand 4.5. The two tabs located on each side of the component are pressed and fitted onto the female components on the socket. There is an additional locating tab, which is used to ensure that the socket orientation is correct. Figure 10 shows the connection of the snap fit clip to the socket collar.

#### Socket structure

The SLS 3D-Printed socket with ABS material was inspired by similar latticed brace designs. An integrated latticed socket, which was created to fit into the designed collar, was able to provide an improved level of breathability for the lower forearm, further light weighting and an aesthetically pleasing design. The breathability and light weight of the socket allows for comfort, and decreases irritation. A decrease in irritation and breathability allows for a decrease in EMG noise.

Due to limitations experienced with the parametrically-based CAD platforms, a cutting-edge new implicit software platform, called nTopology (New York, USA), was used to carry out the socket design.

In order to produce the 3D printed socket, which can be seen in Fig. 13, the pilot's arm was 3D scanned and an STL file generated. The STL file was imported into nTopology where it was possible to optimize the structure. The import allowed for a CAD model to be generated and shelled to create the socket body. The lattice was then defined on an implicit model and lastly various Boolean functions were utilised in order to achieve the final design. The model was therefore converted to an STL file to allow it to be manufactured.

**Fig. 11:**
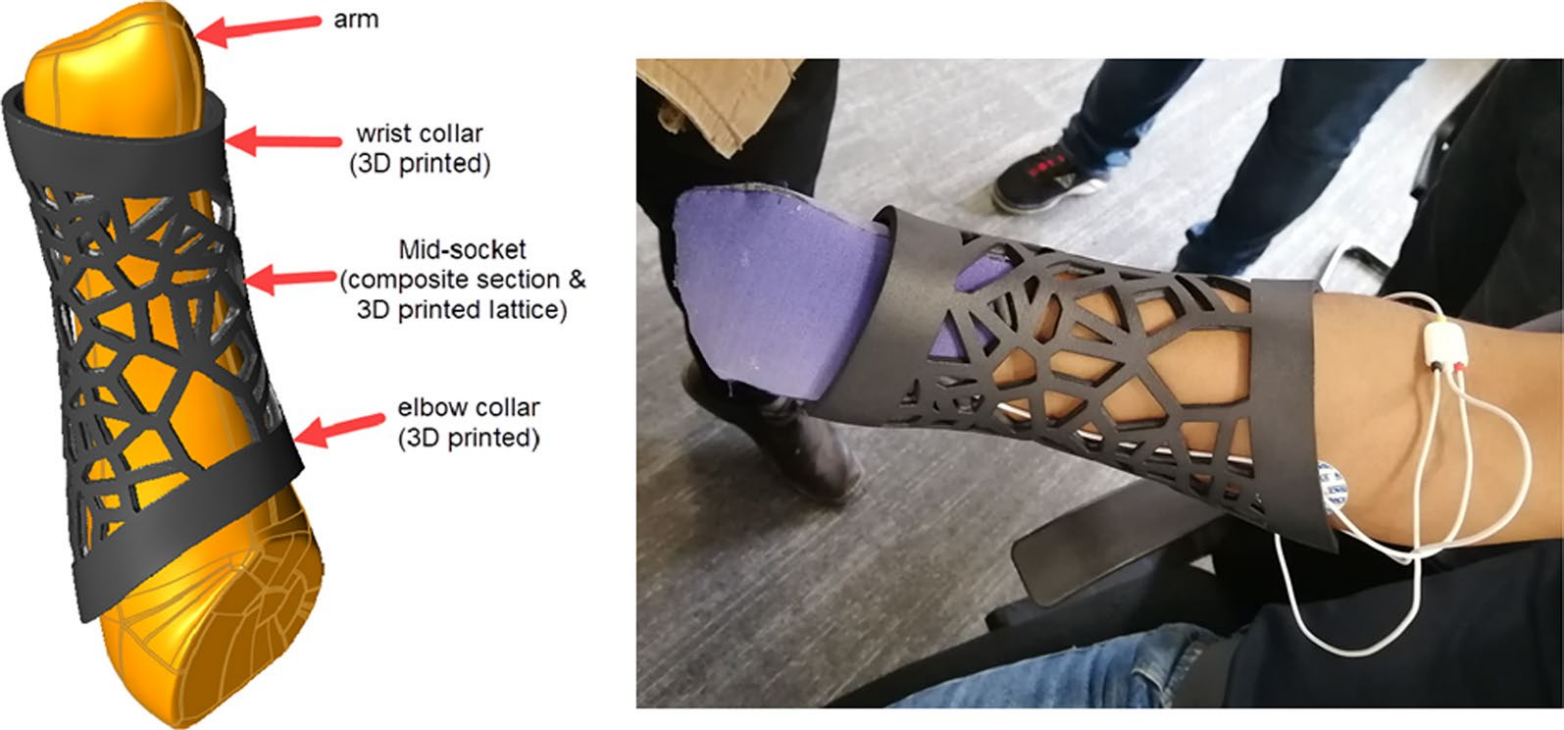
Construction of the 3D printed socket structure

Once the socket structure had been manufactured, the latticed socket was tried on by the pilot, as seen in Fig. 11.

The iteration included updated collars with integrated fastening devices and the lattice structure, which was thinned and cleaned up considerably. The final weight of the optimised lattice socket was 930 g.

Once all the elements of the hand, wrist and socket structure had been manufactured and assembled, the testing of the Touch Hand 4.5 was conducted.

### Electronics design

**Fig. 12:**
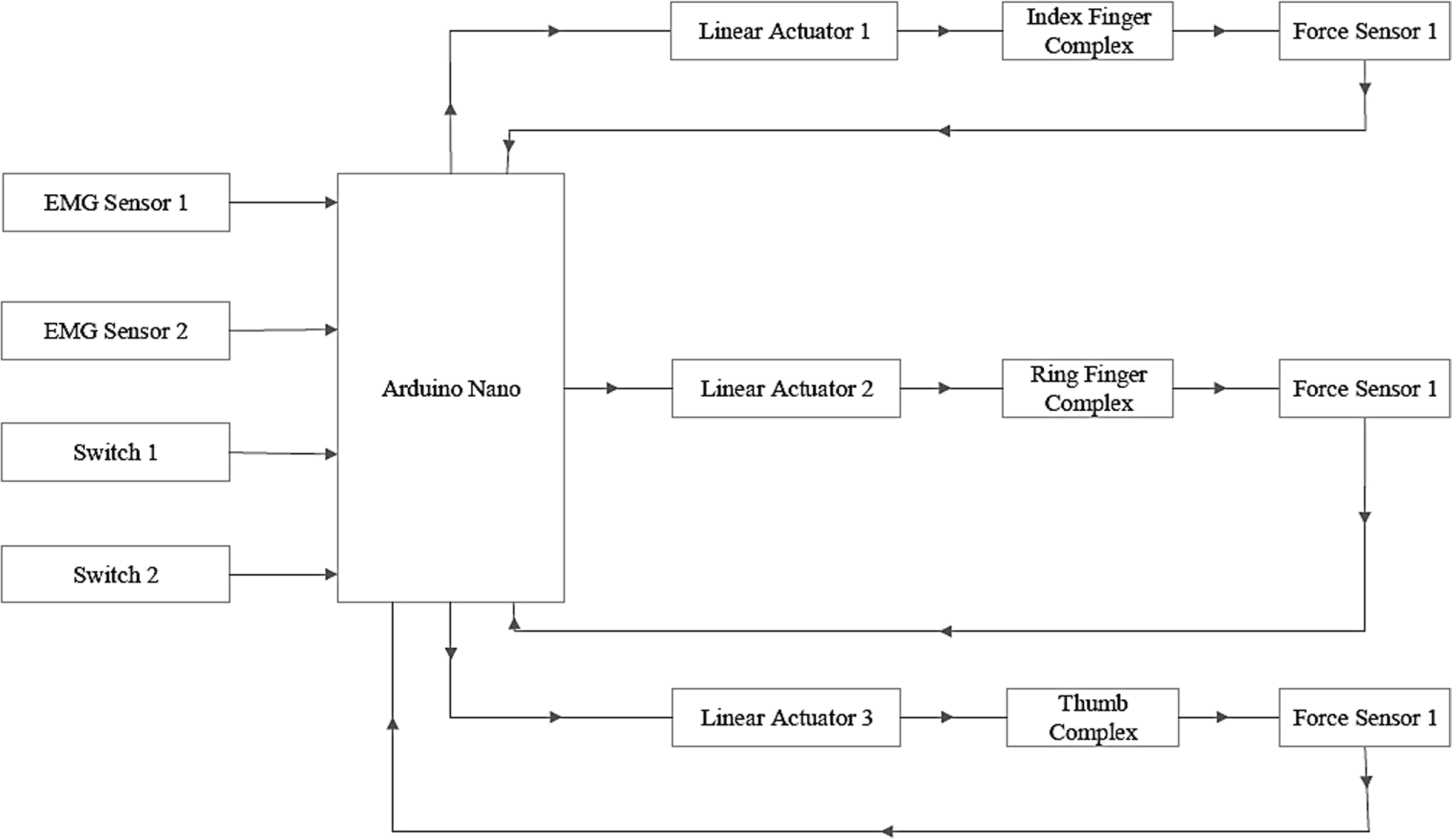
Block Diagram representing the electronics system of the Touch Hand

Figure 12, represents the block diagram of the electronics for the Touch Hand 4.5 prosthetic device:

As seen in the block diagram in Fig. 12, muscle contractions are picked up by the BITalino EMG electrodes (from PLUX – Wireless Biosignals, Lisbon, Portugal) on the arm. The specific muscles that were used to identify the signals, were the forearm muscles, namely the flexor digitorum superficialis and flexor digitorum profundus muscles. These electrodes generate a signal, which is sent to the Arduino Nano microprocessor (from Somerville, Maine, USA). If the strength of the received signal is above a set threshold value, the microprocessor sends a signal to the Actuonix PQ12-30–12-P miniature electric linear motors (from Actuonix Motion Devices, Victoria, Canada), controlling the fingers, to open or close the hand.

This threshold was implemented to prevent accidental opening or closing of the hand due to involuntary or minor muscle contractions and elaborated in the software design section. The signal sent by the microprocessor to the actuators can be intercepted by switches connected to the ring and index fingers. These switches can be turned on and off irrespective of each other to allow different grips. Once the linear actuator receives the instructions, the thumb and finger complexes actuate accordingly, as per the EMG signals received from the flexor digitorum superficialis and flexor digitorum profundus muscles. The readings of the FSR400 force sensors (from Interlink Electronics, Los Angeles, California, USA), which are below the finger silicone covers, are processed and sent back to the motor control system for interpretation. If the set threshold for the force sensor is exceeded at any time during the operation of the hand, the signal to the motors will be interrupted and the fingers will stop actuating.

There are three switches located on the control box, which is worn on the upper arm of the pilot. The switch, which is located in the middle of the switch pad is an emergency stop or power switch. This switch is required to be switched on before the hand is able to operate.

The remaining two switches are connected to the power wires that actuate the fingers. The one is connected to the index finger and the other to the ring finger. By switching these switches on or off, the user can control which fingers open and close independently. When one or both of the switches are switched off, the signal does not reach the actuator and therefore the chosen finger will not respond to any signal from the Arduino. The finger will only respond to a signal if the respective switch is turned on. The thumb does not have a controlling switch and actuates when any signal from the Arduino is received, as long as the power switch is turned on.

### Software design

**Fig. 13:**
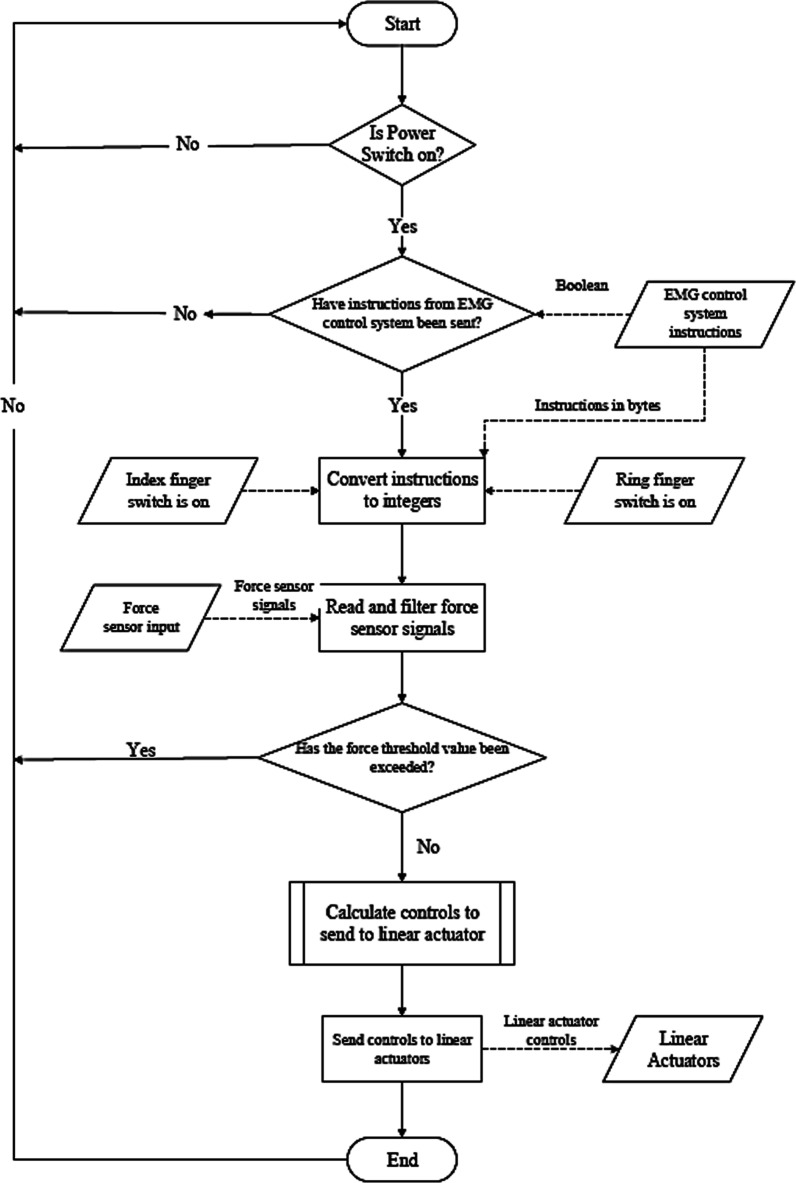
Code flow diagram for the control system of the Touch Hand 4.5

The code flow diagram represents an analysis of the logic behind the data flow of the control system program. There are many different subsystems integrated into the control system of the Touch Hand 4.5. The control system features the microprocessor, the force sensors, and linear actuators. Signals are picked up by the EMG electrodes and processed in each of these subsystems before being interpreted and transferred to the relevant subsystem. The logic flow chart for the entire Touch Hand 4.5 control system is presented in Fig. 13. The sensors are continuously monitored for different threshold values, for the actuators to respond accordingly, depending on the grip that has to be performed. The force sensors and EMG electrode thresholds were identified by a training process with the amputee, to identify the optimal values to be considered depending on the grip they need to perform. The training process is crucial, as different muscle structures from different amputees will differ, therefore different levels of signals will be detected.

## Results

The socket was placed with EMG electrodes on the amputee. Through verbal feedback from the amputee, it was reported that there was less discomfort and irritability due to the breathability of the socket, which was a result of less sweat accumulation between the socket and the amputee’s skin. A comparison was conducted between the lattice 3D printed socket design and the conventional carbon fibre and stainless-steel socket design, which made use of a cloth liner and was initially used by the pilot when testing the Touch Hand 4.5. The amputee reported that he was able to wear the socket and perform tests in preparation for the Cybathlon, for longer periods of time, when compared to the other sockets the amputee had in his possession. The pilot wore the socket for a duration of 4 h, took a break and had it removed for approximately 30 min, and then wore and used the socket for another 4 h.

The Touch Hand 4.5 was designed to perform a range of different grips, depending on the positioning of the fingers and the thumb, in order to complete a number of everyday tasks in the Cybathlon 2020 competition. The silicone finger tips decreased the slippage of items that were picked up, and the force sensor feedback control allowed for sufficient pressure to be applied to the objects, as per the calibrated values. The maximum force between a finger and thumb was 30,4 N, yet the forces were limited with the software and the force sensors depending on the calibrated user and task requirements.

### The pinching grip

**Fig. 14:**
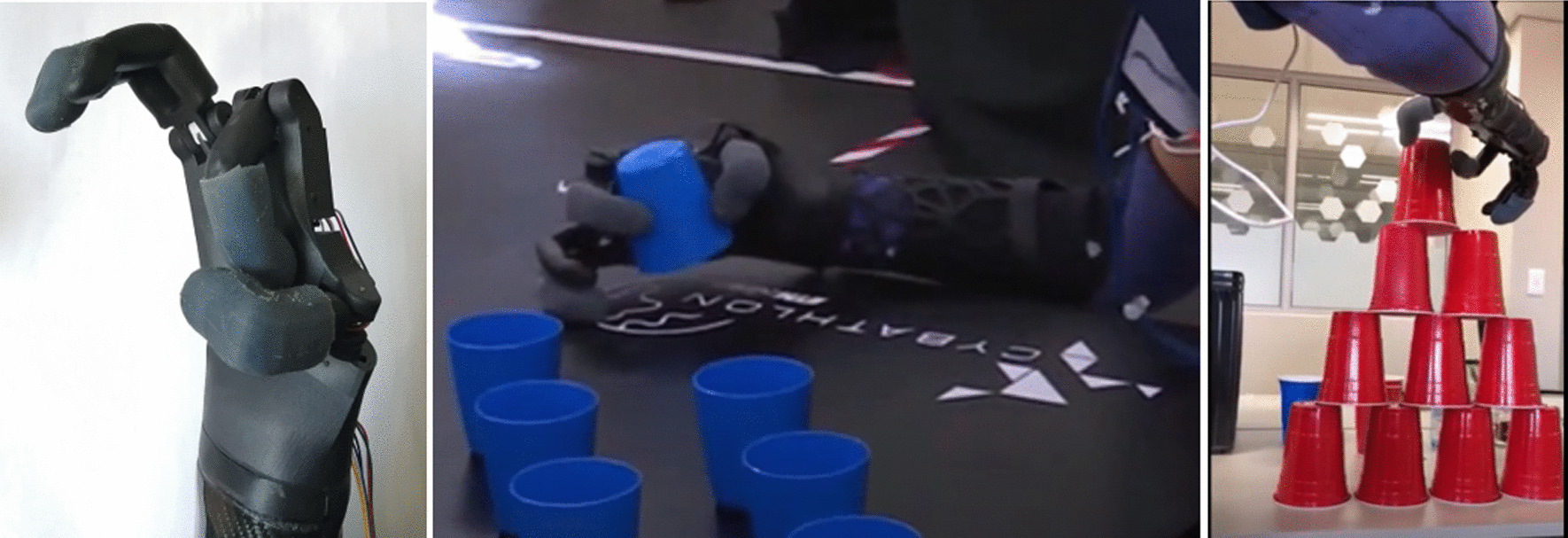
Touch Hand 4.5 demonstrating the pinch grip

**T**he pinching grip is activated when only the index finger and thumb are being actuated, as seen in Fig. 14.

The pinching grip was used to complete tasks that required a high level of precision and accuracy. This grip was used in the Stacking task as the pilot was able to have more control over the cups when picking up and placing the cups into the pyramid form. The Stacking task results are not discussed in detail, even though if more time allowed, this task would have been possible to complete.

### The full grip

**Fig. 15:**
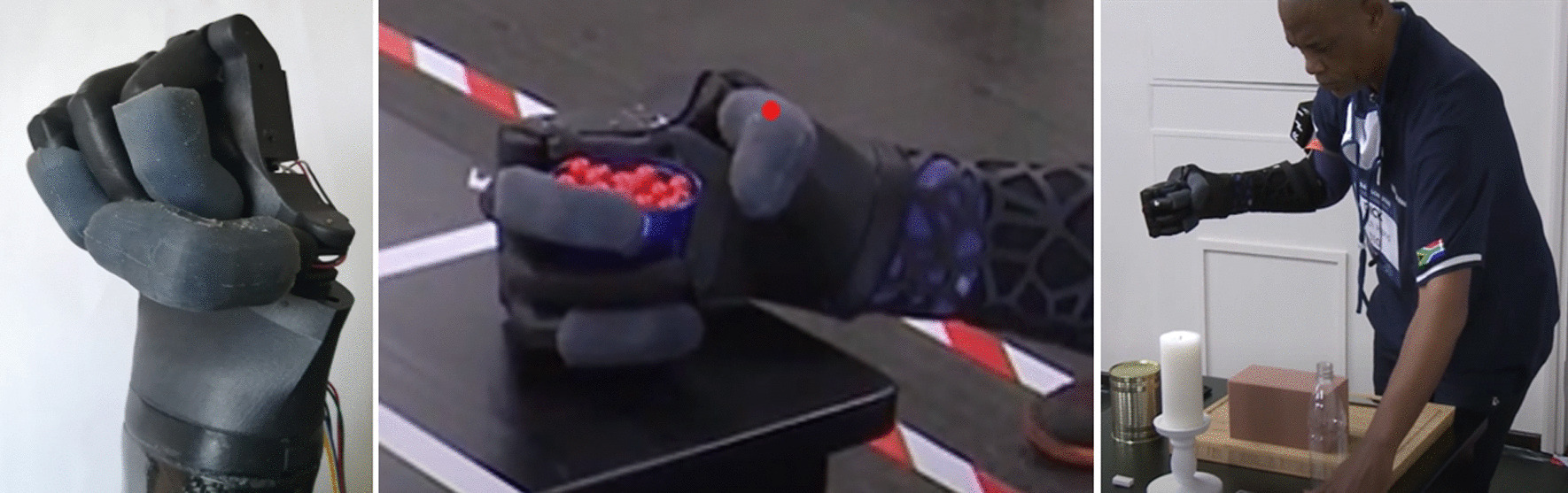
Full grip demonstrated by the pilot using Touch Hand 4.5

The full grip is activated when all the fingers are being actuated, which can be seen in Fig. 15.

The full grip was used in a number of tasks to complete a wide range of activities. This grip was used to picks up and hold objects, such as cups, bottles and glass jars. This can be observed in the Breakfast and Clean Sweep Tasks. This grip also assisted the pilot with holding objects in place, for example holding down a loaf of bread in order to slice it.

### The half grip

**Fig. 16:**
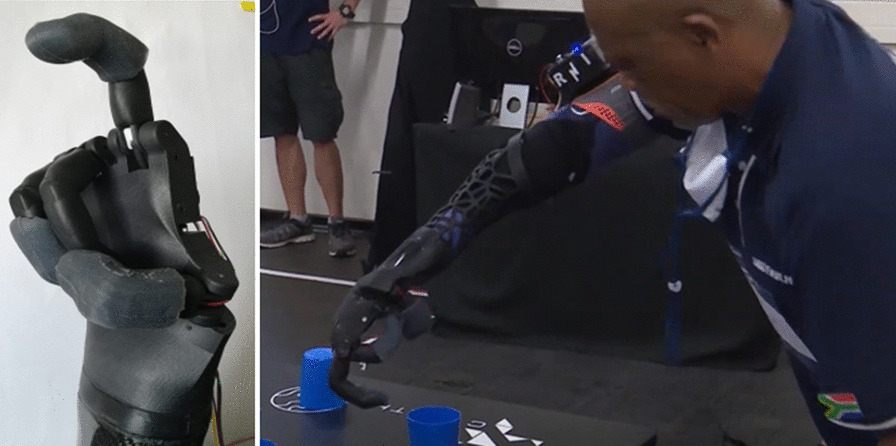
Pilot demonstrating the half grip using the Touch Hand 4.5

The half grip is activated when only the ring finger and thumb are being actuated, which can be seen in Fig. 16.

The half grip was used during the stacking task to move and position the cups and prepare the cups to be picked up and placed into formation. This grip is useful because it allows the index finger to not interfere when moving objects.

### Thumb grip

**Fig. 17:**
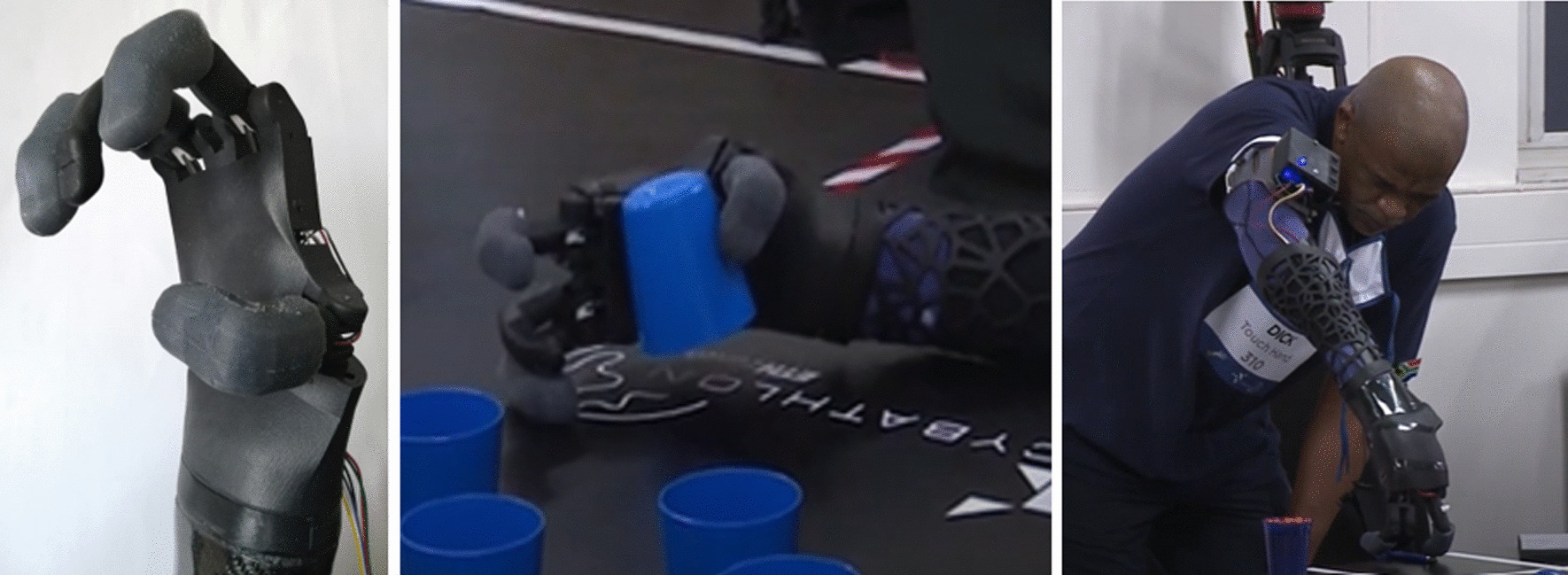
Pilot using the thumb grip to complete different tasks in the Cybathlon 2020 event

This grip is activated when only the thumb is being actuated and can be seen in Fig. 17.

This grip was used during the Clean Sweep task in order to grip small objects, such as the pen, between the index and ring finger complex. This grip was also used during the Stacking task to pick up and flip the cups before they could be stacked.

### Cybathlon results

The Touch Hand team pursued and completed the Breakfast and Haptic Box tasks due to strategy and the competition time limit. These two specific tasks are explained below:

**Breakfast:** The first task in the event is breakfast. This task challenges the pilot’s hand strength and coordination of hands. In order to complete the task, the pilot has to simulate cutting a loaf of bread, unwrap a packet of sugar cubes, open a jam jar, bottle and tin can and light a candle.

**Fig. 18:**
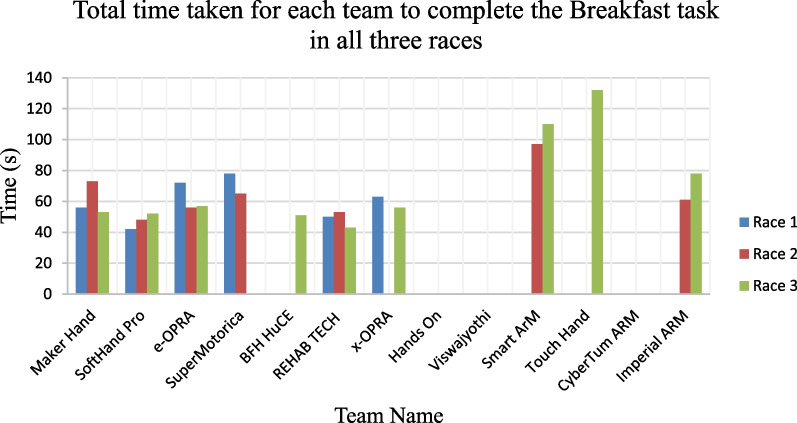
Graph showing the time taken for each of the participating teams to complete the Breakfast task in all three races

**Haptic Box:** The haptic box tests the ability of the prosthetic to feel. The pilot must identify an object in a closed box by touching the object without any visual feedback from the object.

he objects were made from either wood and sponge n the shapes of a block, cylinder and ball. The Touch Hand team, from South Africa, placed eleventh in the ARM discipline of the Cybathlon 2020, out of the thirteen teams that participated in the event. Figure 18 represents the time taken for each of the teams to complete the Breakfast task in all three races.

**Fig. 19:**
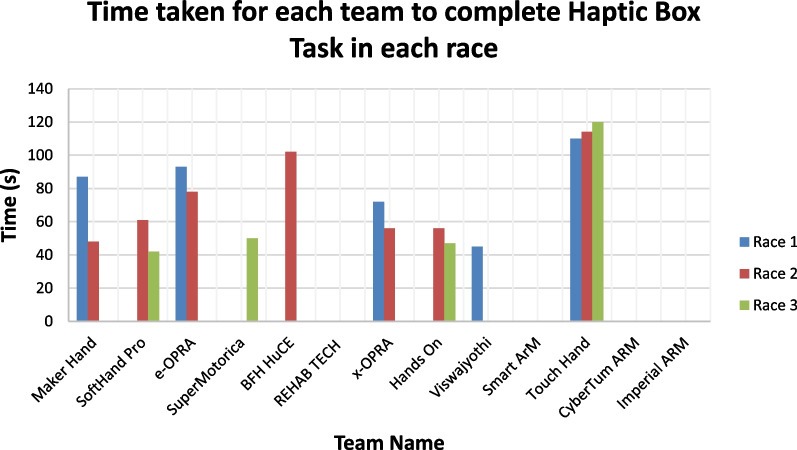
Graph showing the time taken for each team to complete the haptic box task in each race

Figure 19 represents the time taken for each team to complete the haptic box task in each of the three races.

## Discussion

The Touch Hand team took the longest amount of time, in their best race, to complete both the Breakfast and Haptic Box tasks. The times achieved by Touch Hand were significantly higher in comparison with the other teams which managed to complete these Tasks. This indicates that even though the team was able to complete the tasks, an improvement with respect to the time taken to complete the tasks is required. This means that the Touch Hand 4.5 is functional with respect to the hand strength and coordination of the prosthetic hand, which were the aspects assessed by this task, however the device did not perform as well as the other devices competing in the event. This time improvement would be possible to be achieved with faster actuators, and more training and usage of the hand with the pilot. The pilot was able to test the Touch Hand 4.5 for the first time, two weeks prior to the Cybathlon event. Further improvements with the EMG signal decoding methods could be explored, so that the switching for different grips are not needed, which will reduce the time to perform the tasks.

It can be seen in Fig. 18 that the Touch Hand team, along with eight other teams, was able to complete the Breakfast Task in Race 3. The Touch Hand team was only able to complete the Breakfast Task within 132 s in Race 3. There was a 90 s difference between the time the Touch Hand took to complete the task, compared to the best time taken to complete the task, which was achieved by SoftHand Pro. The difference in the time taken to complete the Breakfast Task can be attributed to difficulties experienced with the equipment supplied to the team during the race. Touch Hand team was unable to complete this task in the first two races due to a faulty can opener. A different can opener with a different configuration was used in Race 3, which the pilot had not prepared to learn how to use it, and as a result took much longer to complete this task. This improvement in time could also be achieved if the pilot has had more time to become familiar with the prosthetic device, therefore becoming more confident in using it. The pilot was a little reluctant as to whether different tasks were possible to be achieved with the Touch Hand 4.5, yet with the pilot being persistent and where more time allowed, the pilot was able to find a way to complete the tasks. The time restriction of the races caused the pilot to complete the tasks in a rush, resulting in some panic, and in turn caused the pilot to make mistakes.

The Touch Hand team was the only team who was able to complete the Haptic Box Task in all three races, which can be seen in Fig. 19. Although this team managed to achieve the highest completion rate in this task, they took the longest period of time, on average, to complete this task, which indicates that there is room for an improvement with respect to the performance of the Touch Hand 4.5 prosthetic device. The duration to complete the races were between 100 and 120 s. The pilot indicated that the completion of the task with success was more important than rushing the task. The Haptic Box Task was one of the more complex tasks in the Cybathlon, as this task required the use of the sensory system to determine which object was a match to the object in the box. The Touch Hand 4.5 was able to consistently complete this task because it focused on the sense of touch which most of the prosthetic devices were not able to satisfactorily replicate in their design. Even though there was not actual touch sensory within the Touch Hand 4.5, the pilot indicated that there was a good integration between their arm and the hand, due to the socket fitment, allowing him to feel the motion of the hand as it glided over the object. The pilot indicated that the identification of a hard or soft structure was possible to be identified by the amount of squeeze the hand was able to achieve around the object, and therefore the duration it took for the hand to stop closing.

### Future considerations

The only part of the Home Improvement task that was not possible to be achieved by the Touch Hand 4.5, was the screwing in of the light bulb. The height of the hand was too high to allow for gripping of the bulb and to fit inside of the narrow lamp shade. Therefore, the future design needs to consider a narrower shape to allow for getting into small spaces.

All the other tasks and sub-tasks for the Cybathlon 2020 event were possible to achieve when there were no time restrictions, yet a strategic approach was pursued to obtain the most points (which were assigned to the Haptic Box task), and to attempt the tasks which were possible to achieve in the least amount of time.

## Conclusion

The Cybathlon event has created an international platform, which allows participants from around the world to further advance the technology and design of prosthetic devices. The first contribution that was achieved was the design and development of an additive manufactured hand and socket, considering the socket to have comfort, breathability and the decrease of irritability. A less irritable and breathable socket allowed for a decrease in EMG noise and user-hand unity. The less irritable and breathable socket was achieved with the design optimisation with the use of nTopology. The main issue with respect to upper-limb prosthetic arms is the discomfort and skin irritations users experience when wearing the prosthetic arm. The amputee reported there was less discomfort and irritability with the socket, due to the breathability, as there was a result of less sweat accumulating between the socket and the amputee’s skin. The amputee was able to wear the socket and perform tests in preparation for the Cybathlon, for longer periods of time, when compared to the other sockets the amputee had in his possession.

The second contribution was the design optimisation with the linear actuator integration, for a multi-grip hand, which allowed for the pinch grip, full grip, half grip, and a thumb grip. Slippage prevention with grip force control system integration was also implemented. The pilot also indicated that there was a good integration between their arm and the prosthetic hand, due to the socket fitment, allowing him to feel the motion of the hand as it glided over the object. The pilot indicated that the socket and hand felt more secure and as a part of their body while operating. This integration allowed for the Touch Hand team to be the only team to complete the Haptic Box task successfully.

Due to the time constraints of the ARM discipline of the Cybathlon event, the Touch Hand team was not able to complete the Laundry, Stacking, Clean Sweep and Home Improvement tasks. As a result, the results and data from these four tasks were not used in the analysis and comparison of the devices in the event. The Touch Hand team was the only team to complete the Haptic Box task in all three races, with a duration between 100 and 120 s. The Breakfast task was completed only in Race 3, with a duration of 132 s, due to a faulty can opener that was identified.

## Data Availability

Not applicable.

## Abbreviations

ARM: Powered arm prosthesis race
EMG: Electromyography
CAD: Computer-aided design
CNC: Computer numerical control
3D: Three-dimensional
SLS: Selective laser sintering
ABS: Acrylonitrile butadiene styrene
STL: Standard tessellation language
